# Implementation of streamlining measures in selecting and prioritising complex cases for the cancer multidisciplinary team meeting: a mini review of the recent developments

**DOI:** 10.3389/frhs.2024.1340320

**Published:** 2024-03-12

**Authors:** Tarek Al-Hammouri, Ricardo Almeida-Magana, Tayana Soukup, Benjamin Lamb

**Affiliations:** ^1^ Department of Urology, University College London Hospitals NHS Foundation Trust, London, United Kingdom; ^2^ Department of Urology, The Specialty Hospital, Amman, Jordan; ^3^ Department of Surgery and Cancer, Imperial College London, London, United Kingdom; ^4^ Barts Cancer Institute, Queen Mary University of London, London, United Kingdom; ^5^ Department of Urology, Barts Health NHS Trust, London, United Kingdom

**Keywords:** cancer treatment, health care quality, standard of care, outcome assessment, shared decision making, multidisciplinary team, multidisciplinary team meeting, tumor board

## Abstract

In January 2020, NHS England and NHS Improvement, in the United Kingdom, issued a permissive framework for streamlining cancer multidisciplinary (MDT) meetings. Streamlining is defined as a process whereby complex cases are prioritized for full discussion by an MDT in an MDT meeting (MDM), while the management of straightforward cases is expedited using Standards of Care (SoC). SoC are points in the pathway of patient management where there are recognized guidelines and clear clinical consensus on the options for management and should be regionally agreed and uniformly applied by regional Cancer Alliances. While this report marks the first major change in cancer MDT management since the Calman-Hine report in 1995, its implementation, nationally, has been slow with now nearly four years since its publication. It is argued however that streamlining is a necessary step in ensuring the viability of MDT processes, and therefore maintaining patient care in the current socioeconomic context of rising workload and cancer incidence, financial pressures, and workforce shortages. In this mini review, we offer a succinct summary of the recent developments around the implementation of the 2020 streamlining framework, including challenges and barriers to its implementation, and the potential future directions in this field, which we propose should increase utilisation of implementation science. We conclude that ensuring successful implementation of the framework and the SOC requires securing a buy-in from key stakeholders, including MDTs and hospital management teams, with clearly defined (a) management approaches that include triage (e.g. through a mini MDT meeting), (b) assessment of case complexity (something that directly feeds into the SOC), and (c) roles of the MDT lead and the members, while acknowledging that the SOC cannot be universally applied without the consideration of individual variations across teams and hospital Trusts.

## 1 Introduction

Multidisciplinary team (MDT) meetings are an essential part of cancer care, bringing together healthcare professionals from different disciplines (e.g., oncologists, surgeons, cancer nurse specialists, radiologists, pathologists, physicians, and in some cancers allied health professionals) to discuss patient cases, review diagnostics and develop treatment recommendations (1). However, MDT meetings can be time-consuming and resource-intensive, particularly when discussing straightforward cases that do not require true multidisciplinary input (2). This is further compounded by rising cancer incidents, staff shortages and financial pressures on healthcare (3, 4). Accordingly, there is a need to prioritize complex cases, which are those known to benefit most from a multidisciplinary approach. Indeed, focusing on complex cases has been recommended in several UK national policy documents in recent years (1, 5).

Streamlining is a process whereby complex cases are prioritized for full discussion, while the management of straightforward cases is expedited using Standards of Care (SoC; 4). SoC are points in the pathway of patient management where there are recognised guidelines and clear clinical consensus on the options for management and should be regionally agreed and uniformly applied by regional Cancer Alliances (6). By streamlining cases listed for cancer MDT review, healthcare professionals can work towards improving the efficiency and effectiveness of MDT meetings while still providing high-quality care for cancer patients (3, 7). This review will explore the implementation of the 2020 streamlining framework, emphasizing the vital role of implementation science and the importance of securing buy-in from key stakeholders.

## 2 The 2020 streamlining framework and its implementation

The guidance from NHS England (5) marks a departure from the NHS directives of the past 20-years (8). It suggests that not all cases require discussion and that the focus should be on complex cases. This provides cancer MDTs with a clear mandate to implement changes. The question of what constitutes a complex case, is however not so readily answered in the guidance. This question is an important one, as failure to streamline MDT processes using existing best evidence means that while the team's caseload may become more manageable, the care quality could be compromised by returning to the unwarranted variation in care that was evident before the introduction of MDTs (9, 10).

What constitutes the complexity of a cancer case for MDT discussion has been addressed scientifically and concurrently with the NHS England guidance by Soukup and colleagues, who spent 2-years undertaking an NIHR-funded mixed methods study, with input from hundreds of cancer experts, and data from hundreds of cancer MDT case discussions across the UK (11). The researchers found that each professional group within an MDT holds a unique perspective on the question of what constitutes a complex case. Their algorithm, Measure of Case-Discussion Complexity: the MeDiC tool, includes 26 psychometrically validated indicators of complexity that represent the perspectives of all professional groups that make up an MDT. This tool allows MDTs to scientifically measure case complexity and apply this to streamlining and the selection of cases for the SoC. Subsequent research demonstrated feasibility and utility of the MeDiC tool in urology MDTs (12).

Implementing the streamlining guidance, along with structuring and organizing the MDT meeting, will require time and effort, especially if using scientific tools such as MeDiC. What resource is required, and how this is best utilized is open to debate, and the optimal strategy will likely vary from one team to another. It is hoped however, that this investment will pay dividends by allowing better utilization of these resources by focusing on complex cases, and reducing unnecessary delay for cases that meet SoC (7).

In addition, several models have been proposed to facilitate triage of cases referred to the MDT meeting, and efficient decision-making and patient management (9, 13).

The first model, referred to as the Mini MDT, constitutes a core team comprising the MDT Coordinator, MDT lead (or deputy), radiologist, and pathologist. Within this framework, all cases are subject to discussion within the Mini MDT (14). The Mini MDT collectively evaluates the results of investigations and decides whether a case should be referred for SoC management or necessitates full MDT discussion. Cases designated for SoC management have their management recommendations meticulously documented, while MDT cases undergo comprehensive deliberation during the subsequent full MDT meeting, involving the complete team.

The Pre-MDT triage model (15), on the other hand, adopts a different approach. It involves a smaller triage team, consisting of the MDT Lead (or deputy) and the MDT Coordinator. For a case to be considered under this model, the radiology and pathology reports must have been reviewed and reported by a core MDT member. The pre-MDT triage team systematically reviews all cases and makes determinations regarding their categorization, either for SoC management or full MDT discussion. As with the previous model, SoC cases have their management recommendations meticulously documented and integrated into the MDT minutes, while MDT cases are subsequently subjected to comprehensive examination during the full MDT meeting, where the entire team contributes to the discussion.

A third suggested model places emphasis on active engagement from all clinicians involved in the MDT (13). The prerequisite, once again, is that radiology and pathology reports must have been reported by a core MDT member. Under this model, referring clinicians take on the responsibility of assessing whether a case warrants SoC management or necessitates full MDT discussion. The referring clinician then documents the management recommendation accordingly. Subsequently, the MDT lead reviews both the SoC and MDT lists to ensure the appropriate categorization. Cases designated for full MDT discussion are deliberated upon in detail during the MDT meeting, involving the complete team.

These three models for MDT triage enable the allocation of appropriate resources and the identification of cases that necessitate comprehensive MDT discussion. By adhering to specific criteria and actively involving relevant team members, these models facilitate streamlined decision-making processes, ultimately ensuring the delivery of optimal patient care outcomes (16).

In this process, the role of the MDT lead holds significant importance. To ensure that the individual appointed for this role possesses the requisite experience, interest, and credibility, a competitive interview process should be considered (17). This interview process serves to assess the candidate's qualifications and suitability for the position. Furthermore, it is imperative that the role of the MDT lead is clearly defined, with a well-defined description outlining the responsibilities and expectations associated with the position (13). To ensure effective execution of the MDT, members should cover various essential activities listed in Table 1.

**Table 1:** Collaborative roles and responsibilities within multidisciplinary team (MDT) operations.

Role of the MDT Lead	Role of other members
- Necessary oversight: Providing guidance and coordination for MDT activities.	- Allocate appropriate time within their job plans
- Preparation for meetings: Reviewing patient information, conducting research, and collecting data for informed MDT discussions.	- Collectively agree upon their relative contributions to the preparation for these meetings
- Participation in improvement efforts: Actively contributing to enhancing MDT efficiency and effectiveness.	
- Implementation of streamlining: Identifying opportunities to optimize workflow and improve team performance.	
- Maintenance of audit: Ensuring accurate documentation of MDT decisions and monitoring outcomes.	

The roles within MDTs, as detailed in Table 1, are pivotal to the streamlining and efficiency of cancer care, yet their effective implementation necessitates adaptability to the unique environments of various teams and hospital trusts. The MDT Lead plays a crucial role in providing necessary oversight, preparation for meetings, participation in improvement efforts, implementation of streamlining strategies, and maintaining audits. This role, however, must be flexible enough to accommodate the diverse challenges and resources of different healthcare settings. For instance, in smaller trusts, the MDT Lead might engage more directly in the preparation and review of patient information, while in larger settings, their focus might shift towards strategic coordination and oversight of streamlining efforts.

Similarly, the roles of other team members, who contribute to meeting preparation and actively participate in streamlining and improvement initiatives, must be attuned to the specific dynamics of their team. In some settings, team members might allocate significant time to the detailed preparation of cases due to the complexity or volume of patients, while in others, their focus might be on collective efficiency and streamlined decision-making processes. Adaptability in these roles is essential to cater to varying patient loads, resource availability, and organizational structures across different hospital trusts.

By recognizing and adapting to this variability, MDTs can ensure that their roles are not only clearly defined but also flexible and responsive to their specific healthcare environment. This adaptability is key to maintaining high standards of patient care, irrespective of the differing contexts and challenges presented by each trust.

## 3 Challenges, barriers, and the role of implementation science

While efforts to streamline MDT meetings are crucial, the potential disadvantages of implementing streamlining in MDT meetings needs to be considered. The specific disadvantages may vary depending on the context and implementation approach, and some to consider are listed in Table 2. It is important to carefully consider these and design streamlining strategies that strike a balance between efficiency and comprehensive patient care (18). Engaging MDT members, maintaining open lines of communication, and regularly evaluating the impact of streamlining efforts can help address these disadvantages and optimize the benefits of streamlining in cancer MDT meetings (19).

**Table 2:** The complexities and potential drawbacks of streamlining in multidisciplinary team (MDT) meetings.

1. Misapplication of Definitions of Complexity:	
- Streamlining efforts may lead to mismanagement of complex cancer cases if complexity definitions are not accurately applied.	- Incorrect or misapplied definitions can overlook important nuances requiring interdisciplinary discussion.
2. Limited Time for Comprehensive Discussion	
- Streamlining intended to focus on complex cases may inadvertently reduce overall discussion time, including for complex cases.	- Complex cases may require extensive discussions for optimal decision-making.
3. Potential for Biased Decision-Making	
- Streamlining can introduce biases if standardized approaches prioritize certain patient aspects.	- May overlook factors like patient preferences, social circumstances, or emerging treatments, leading to biased decisions.
4. Reduced Interdisciplinary Collaboration	
- Streamlining involving fewer disciplines can save time but limit the benefits of interdisciplinary collaboration.	- Comprehensive understanding and improved treatment planning may be compromised.
5. Incomplete Information and Data Gaps	
- Streamlining relies on accurate, comprehensive patient data, which data gaps can compromise.	- May result in suboptimal decisions or inadequate consideration of patient needs.
6. Lack of Flexibility for Individual Variations	
- Streamlining's one-size-fits-all approach may overlook individual patient variations in characteristics, comorbidities, or treatment responses.	- Impact on treatment outcomes due to lack of individualized care.
7. Resistance from Team Members	
- Implementing streamlining measures may face resistance from team members who perceive it as a threat to their professional autonomy.	- Overcoming resistance and ensuring team buy-in is crucial for successful streamlining.

It is also important to anticipate different barriers to implementation of streamlining, such as for example challenges in selecting and prioritising complex cases (20, 21). Resistance to change is a common hurdle, as implementing streamlining measures often disrupt established routines and roles within the MDT. Additionally, necessary resources are often found to be lacking, including dedicated personnel and technology upgrades. This is improved by engaging various stakeholders, from clinicians to administrators, as disengagement from specific groups can hamper progress (22).

Challenges in the form of communication and coordination within the team can further complicate the process. Given that MDT meetings are already time-limited, time constraints can lead to rushed decision-making when introducing streamlining measures. Furthermore, an efficient streamlining process relies on access to accurate patient data, test results, and treatment guidelines (20). Barriers related to data availability, privacy concerns, or incomplete information can hinder the streamlining process. Finally, institutional culture, existing policies, and governance structures may either facilitate or hinder streamlining efforts, and legal and regulatory considerations must be navigated carefully.

## 4 Strategies for successful implementation

Mitigating the barriers necessitates the adoption of comprehensive strategies (21). It involves effective change-management strategies, adequate resource allocation, stakeholder engagement, and clear communication regarding the advantages of streamlining. Collaboration among MDT members, along with leadership support and commitment to adapt and learn from the implementation process, is key to overcoming these challenges and successfully streamlining cancer MDT meetings.

It is therefore important to consider optimising existing processes in MDT meetings before embarking on significant changes to the standard operating procedures (9, 23). First, a comprehensive assessment and audit of the current local circumstances is imperative, encompassing a meticulous evaluation of case volume, temporal allocation, personnel availability, and their respective contributions (24). Secondly, a revision of the clinical data available for decision-making should encompass comorbidities, social determinants, performance metrics, radiological findings, pathology reports, and patient perspectives where accessible (25). Furthermore, a judicious approach to measuring case complexity should be adopted, and the employment of a structured template or proforma is recommended (26). The team should refrain from discussing cases that could be appropriately managed elsewhere, focusing solely on cancer cases during the MDT meeting.

To facilitate a comprehensive, holistic, and patient-centred care, the recognition and cultivation of good team dynamics, effective meeting management, and the mitigation of disruptive behaviours and distractions are important. Avoiding excessively lengthy meetings and incorporating breaks to refresh participants are also important considerations (27). Finally maintaining representative and transparent record-keeping is crucial.

Lastly, establishing SoC is critical as it requires a clear consensus regarding the most effective care management for patients (28). These standards hinge on several key factors: firstly, the condition must be categorized within the criteria for low complexity, furthermore, patients must meet the eligibility criteria with minimal comorbidities, indicating lower chances of adverse outcomes. Secondly, a robust consensus should exist regarding the optimal management strategies for the specific condition in question (22), while patients' willingness and ability to adhere to the recommended approach is considered. This approach could ensure consistent, safe, and effective management practices for low-risk conditions, ultimately optimizing patient outcomes and judiciously allocating resources.

## 5 Future directions and role of implementation science

Implementation science plays a pivotal role in the streamlining of MDT meetings in cancer care, bridging the gap between established guidelines and their practical application. With its focus on the methods to promote the uptake of research findings into routine healthcare practices, it offers invaluable insights for enhancing MDT meeting efficiency and effectiveness. It provides a structured framework for identifying, analyzing, and overcoming the barriers to successful implementation. By employing implementation science principles (e.g., 29–33), MDT meetings can adopt a more systematic and evidence-based approach to prioritize complex cases, optimize decision-making processes, and adapt to the unique challenges of different healthcare settings. As outlined in the recent paper (7), it equips MDMs with the necessary tools to evolve from traditional, all-encompassing discussions to a more focused and strategic model of patient case review, which is crucial in the current landscape of increasing cancer incidence and resource constraints (7).

Securing stakeholder buy-in is also a critical component in the successful implementation of streamlined MDT meeting processes. Implementation science emphasizes the need for engaging all key stakeholders—from managers to oncologists and pathologists to nurses and administrative staff—ensuring that each voice is heard in shaping the implementation. This inclusive approach not only fosters a sense of ownership among MDT members but also facilitates the identification of team-centred solutions that are sensitive to the unique dynamics and needs of each team. Moreover, implementation science provides the tools and methodologies (e.g., 33) to tailor the streamlining strategies to fit diverse settings, acknowledging that a one-size-fits-all approach is rarely effective. By leveraging these principles, healthcare organizations can develop and implement streamlining strategies that are both effective and sustainable. These tailored strategies not only streamline the decision-making process but also enhance collaboration and communication within teams, ultimately leading to a more agile and responsive cancer care system. Through this lens, implementation science is not just a facilitator for change; it is a catalyst for creating a more dynamic, efficient, and patient-centered MDT model (7).

In addition to these direct applications, a critical aspect of implementation science in streamlining MDT meetings lies in its contribution to building and expanding a knowledge base. As MDTs adopt these streamlined approaches, the collection, analysis, and dissemination of data on their implementation and clinical effectiveness will become invaluable. This ongoing process of knowledge creation not only informs the refinement of current practices but also serves as a rich resource for other teams embarking on similar streamlining journeys. By systematically documenting successes, challenges, and lessons learned, a robust body of evidence can be generated. This evidence base is essential not only for continuous improvement within individual teams but also for advancing the overall practice of cancer care. It supports the development of best practices that can be shared and adapted across different contexts, further enhancing the capability of MDTs to provide high-quality, efficient, and patient-focused care in an ever-evolving healthcare landscape (7, 29–33).

## 6 Conclusion

The workload of MDTs is on the rise, while the effectiveness of MDT processes exhibits variability. To enhance effectiveness and efficiency, streamlining measures need to be implemented. It is crucial to concentrate the MDT meetings on complex cases, as these often require comprehensive interdisciplinary collaboration. Successfully implementing SoC necessitates directing attention towards several key factors, including areas of consensus, complexity of cases, local agreement, the operational model, and acknowledging that SoC cannot be universally applied without consideration of individual variations. Looking ahead, the integration of implementation science principles will be crucial in adapting and evolving the streamlining practices to meet the diverse needs of cancer care.
